# Endovascular repair of the aortic arch in pigs by improved double-branched stent grafts

**DOI:** 10.1308/003588413X13511609955814

**Published:** 2013-03

**Authors:** C Lin, L Wang, Q Lu, C Li, Z Jing

**Affiliations:** ^1^Department of General Surgery, Fuzhou General Hospital, Fuzhou, China; ^2^Department of Vascular Surgery, Changhai Hospital, Shanghai, China

**Keywords:** Endovascular repair, Stent graft, Aortic arch, Branch, Pig

## Abstract

**Introduction:**

This study aimed to evaluate the feasibility of total endovascular repair of the aortic arch in pigs using improved integrated double-branched stent grafts.

**Methods:**

Improved self-expandable stent grafts with a main body and two integrated branches were prepared for the repair of the aortic arch in six pigs. The feasibility of using these stent grafts was evaluated with arteriography, computed tomography (CT), computed tomography angiography (CTA) and autopsy three months following the procedure.

**Results:**

The double-branched stent grafts were placed successfully in the aortic arch in all six pigs. All pigs survived for at least three months and their biological behaviour was normal. Arteriography, CTA and animal necropsy revealed good fixation in all cases. Aortic valve function and coronary ostia remained intact, and CT of the head did not detect any lesion of cerebral infarction.

**Conclusions:**

Endovascular repair of the aortic arch with an integrated double-branched stent graft is safe and feasible in animal studies.

Aortic arch diseases including aortic dissection and aneurysm contribute substantially to morbidity and mortality. Currently, surgery remains the main treatment for aortic arch diseases. Despite recent advances in traditional surgical techniques and anaesthetic management, the surgical repair of the thoracic aorta is still associated with significant mortality and morbidity, probably due to related surgical trauma.^1,2^ In recent years, transluminal endovascular stent graft placement has emerged as a promising alternative to surgical treatment of aortic aneurysms. Although endovascular stent grafting is less invasive than open surgical procedures, the involvement of supra-arch branch vessels such as the brachiocephalic trunk (BCT), the left common carotid or the left subclavian artery (LSA) limits the application of stent grafting.

Endovascular management of arch vessels consists of two approaches: hybrid operations and total endovascular repair without open revascularisation of the arch vessels prior to stent graft insertion. The latter approach comprises three techniques: in vivo fenestrated stent grafts, the chimney stent graft technique and homemade fenestrated plus branched stent grafts. Application of these techniques allows a greater number of individuals to undergo endovascular repair of the aortic arch.

We have developed a new method of managing supraarch vessels in which an improved integrated stent graft with side branches for these vessels is employed. This study aimed to evaluate the feasibility of total endovascular repair of the aortic arch using the custom made integrated double-branched stent graft in a porcine model.

## Methods

### Animals

Six domestic hybrid pigs weighing 31–40kg (mean: 35.7kg, standard deviation [SD]: 3.2kg) were provided by the laboratory animal research centre of the Second Military Medical University (SMMU). The study was approved by the ethics committee of the SMMU and all animals received humane care throughout the study.

### Stent graft design

**Figure 1:**
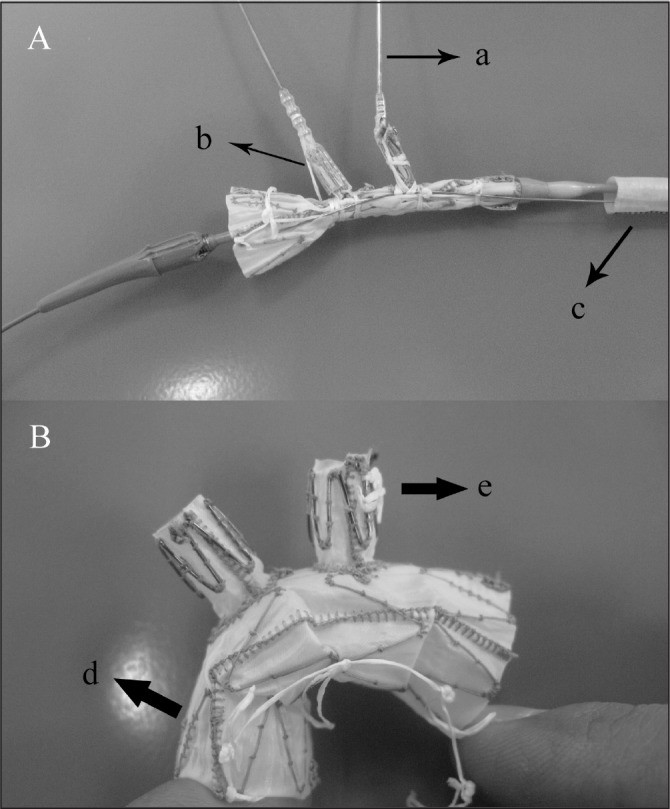
Double-branched stent graft system: the delivery system of the stent graft (A) consisting of an outer sheath, an inner sheath and two traction wires (a = traction wire, b = protective cap, c = inner sheath) and the integrated stent graft in the unfolded state (B) consisting of the aortic section (d) and the branched section (e)

A self-expandable integrated stent graft was designed that consisted of two branches for the two supra-arch branch vessels and a main body for the aortic arch of pigs (Fig 1). The raw material of the stent was nitinol and the graft covering the stent surface was made of polyethylene terephthalate. The branches and main body were sutured with 6/0 Prolene^®^ (Ethicon, Somerville, NJ, US). The thickness of the graft was about 0.1mm. The size of the stent graft was customised based on the size of the target vessels measured via preoperative arteriography. The external diameter of the stent graft was 20% larger than that of the proximal and distal landing zones of the target vessels as such an oversized design was able to achieve better fixation.^3^

### Delivery system

The delivery system consisted of an inner (soft) and an outer (stiff) sheath. The stent graft was compressed evenly and tied with a control wire passing through crossed bundling coils. It was then crimped to fit into the inner sheath. Each branch of the stent graft was folded in a protective cap adhered to the traction wire (Fig 1). The outer sheath gave the whole system a smooth surface.

**Figure 2:**
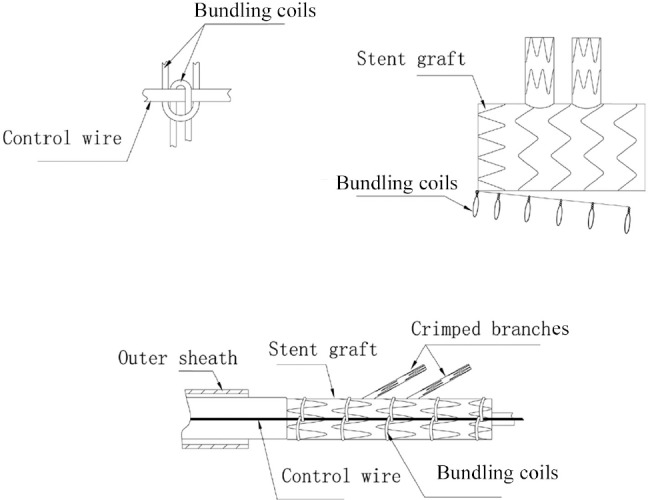
Schematic diagram demonstrating how the bundling coils folding the stent graft were bound by a control wire

On delivery, the outer sheath was pulled back. The system thereby became flexible and was located in the correct position of the aortic arch. The stent graft remained in the folded state when the inner and outer sheaths were both withdrawn, which allowed the adjustment of the in vivo location of the stent graft. Only when the control wire was pulled out, did the bundling coils spontaneously untie themselves, and the stent graft was fully released and inserted (Fig 2). The external diameter of the whole system was 6.09mm (18Fr).

### The triple lumen catheter

**Figure 3:**
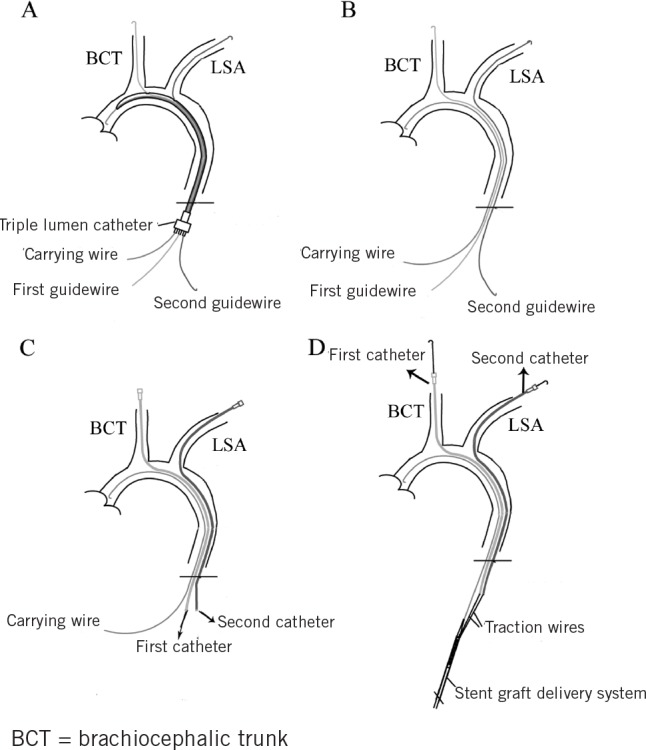
Schematic diagram demonstrating how to prevent wire entanglement by using the triple lumen catheter: The triple lumen catheter was advanced to the aortic arch via a carrying wire, with the first guidewire implanted previously from the right subclavian artery (RSA) in another lumen. The second guidewire was placed in the vessel leading from the infrarenal aorta to the left subclavian artery (LSA) through the remaining lumen of the triple lumen catheter (A). When the triple lumen catheter was removed, the carrying wire and the guidewires remained uncrossed (B). The two guidewires were replaced by two catheters and the carrying wire was changed to a stiff wire (C). The delivery system was introduced into the aorta via the stiff wire. The free ends of the two traction wires were pulled out of the LSA and RSA through the previously implanted catheters (D).

There were three parallel independent lumens in the triple lumen catheter. One was for the carrying wire and the other two for the traction wires of the two branches. The triple lumen catheter prevented the wires from becoming twisted during the procedure (Fig 3).

### Aortography and measurement of target vessels

The pigs were sedated with an intramuscular injection of ketamine hydrochloride (8mg/kg) followed by induction with intravenous pentobarbital (12mg/kg). After sedation and tracheal intubation, an intravenous injection of heparin (100iu/ kg) was administered to each pig and the sheath catheters were inserted in the right femoral artery of the pigs using the Seldinger method. A total of 25ml of contrast agent (Ultravist^®^ 300; Schering, Berlin, Germany) was injected at a rate of 15ml per second via a 5Fr marked pigtail catheter.

### Stent graft deployment procedures

The procedure was conducted in pigs under intravenous pentobarbital general anaesthesia (12mg/kg) and tracheal intubation. The pigs were placed in the dorsal decubitus position, and the infrarenal aorta, RSA and LSA were exposed surgically.

**Figure 4:**
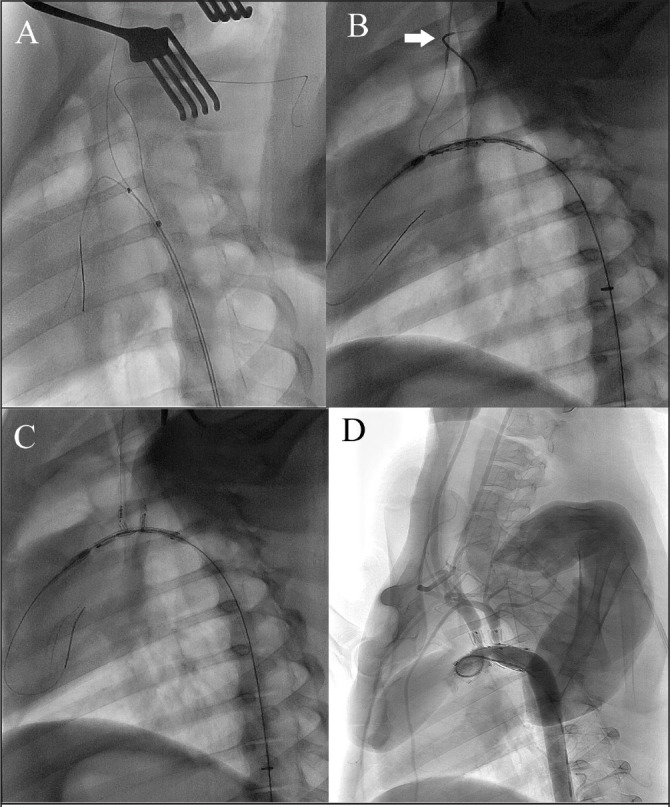
Digital subtraction angiography showing deployment of the stent graft: The triple lumen catheter was advanced into the aortic artery with one carrying wire and two guidewires in its parallel lumens (A). The stent graft reached the aortic arch (B). The branches of the stent graft were pulled into the brachiocephalic trunk and left subclavian artery by the traction wires (C). The stent graft was fully deployed (D).

The pigs were given 4,000iu heparin intravenously before two 6Fr introducer sheaths were inserted into the subclavian arteries. A 0.035” guidewire was inserted through the sheath in the RSA into the infrarenal aorta. A transverse arteriotomy was performed subsequently in the infrarenal aorta and the wire end was pulled out of the incision. Next, a triple lumen catheter was advanced to the aortic arch from the incision via a carrying wire, with the first guidewire previously implanted in another lumen. A second 0.035” guidewire was placed in the vessel from the infrarenal aorta to the LSA through the remaining lumen of ****the triple lumen catheter (Figs 3A and 4A). The triple lumen catheter was then removed and the three wires remained parallel in the lumen of the artery (Fig 3B).

The two guidewires were soon replaced by two catheters and the carrying wire was changed to a stiff wire (Fig 3C). The delivery system was then introduced into the aorta via the stiff wire. The free ends of the two traction wires were pulled out of the LSA and RSA through the previously implanted catheters located in the abdominal aorta (Fig 3D).

When the delivery system reached the descending aorta, the outer sheath was withdrawn but the inner sheath was kept in place so that the delivery system passed the aortic arch smoothly (Fig 4B). After the inner sheath was pulled back, the branches of the stent graft, which remained in a folded state, were drawn into the BCT and LSA (Fig 4C). Once the control wire was pulled out, the branches and main body of the stent graft deployed completely. The protective caps were drawn out of the body together with the traction wires at the end of the procedure. The delivery system was eventually removed and the incisions were closed. Aortography was performed again to verify the fixation of the stent graft (Fig 4D).

### Postoperative treatment

After the operation, the pigs received sodium benzylpenicillin (1.6 million international units, intramuscular, twice daily) for five consecutive days to prevent bacterial infection. They also received an anticoagulation regimen (Aspirin 100mg orally, daily). In some cases, they received a preventive anticoagulation regimen instead, which included three days of low molecular heparin (30mg by hypodermic injection, twice daily) and warfarin (2.5mg orally, daily), starting from the third day after the stent graft deployment. In addition, the pigs received proper intravenous fluid replacement until regular food intake was commenced.

### Evaluation of the stent graft deployment

After implantation of the stent graft, the animals were reared for an additional three months before they were evaluated for biological behaviour such as life habit and limb movements. CT of the head without contrast enhancement was performed to identify potential cerebrovascular infarctions. Arteriography and CTA were used to evaluate the in vivo status of the deployed stent grafts in the third month after the procedure. At the end of three months, the pigs were sacrificed and the thoracic aortas were dissected to examine the fixation status of the stent grafts. The arterial segments in which the stent grafts were placed were sectioned, stained with haematoxylin and eosin, and analysed under light microscopy.

## Results

The details and outcomes of the stent graft deployment are shown in Table 1. The procedure was successful in all six pigs. The mean operation time was 110.8 minutes (SD: 12.1 minutes). In every pig, postoperative aortography confirmed that the aortic arch and its two main supra-arch branches were free of any obstruction and no malattachment was found.

**Table 1:** Profiles of the stent graft deployments in six pigs

Sex	Preoperative weight	Postoperative weight*	Diameter				Operation time
			BCT	LSA	PLZ	DLZ	
1 Female	40.3kg	49.8kg	6.51mm	6.48mm	12.9mm	11.3mm	102 mins
2 Male	34.2kg	45.5kg	5.27mm	5.28mm	13.6mm	12.1mm	116 mins
3 Female	31.8kg	39.7kg	5.02mm	5.00mm	11.8mm	10.9mm	126 mins
4 Male	35.8kg	45.2kg	6.05mm	6.02mm	13.9mm	12.4mm	97 mins
5 Male	38.3kg	49.5kg	6.11mm	6.10mm	12.7mm	12.0mm	102 mins
6 Female	33.6kg	42.1kg	5.72mm	5.72mm	12.3mm	11.5mm	122 mins

BCT = brachiocephalic trunk; LSA = left subclavian artery; PLZ = proximal landing zone; DLZ = distal landing zone

* three months after the procedure

The pigs were followed up for three months and their average weight increased from 35.7kg (SD: 3.2kg) to 45.3kg (SD: 4.0kg). Their postoperative digestive function and biological behaviour remained normal without any sign of motor function deficits, dysarthria, dysphagia or claudication. CT of the head without contrast enhancement indicated no evidence of cerebral infarction.

**Figure 5:**
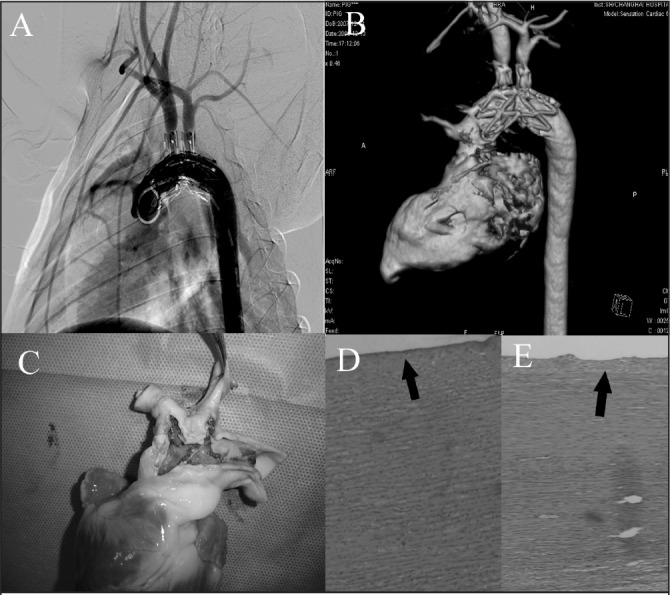
Evaluation of the stent graft deployment: postoperative angiography showing that the coronary artery and the two branches of the aortic arch were unobstructed (A), computed tomography angiography showing the double-branched stent graft in the aortic arch (B), animal necropsy showing the secure fixation and sealing of the stent graft (C), and haematoxylin and eosin staining (50x magnification) showing the intact intima in the brachiocephalic trunk and left subclavian artery (D and E)

Three months after the operation, arteriography and CTA confirmed the immobilisation of the double-branched stent graft in every pig (Figs 5A and 5B). None of the pigs developed backflow through the aortic valve, and their valve functions and coronary arterial ostia appeared uncompromised.

After the deployment, all blood supply to the brain and upper extremities passed through the branches of the stent graft, which were the narrowest part of the stent graft with an internal diameter of no more than 7mm. To assess whether blood supply to these areas was sufficient, the pre and postoperative arterial blood pressure was measured in the BCT and LSA in the third month after stent graft deployment. The results are shown in Table 2.

**Table 2:** Mean arterial pressure in pigs’ brachiocephalic trunk (BCT) and left subclavian artery (LSA)

	BCT pressure		LSA pressure	
	Preoperatively	Postoperatively*	Preoperatively	Postoperatively*
1	120mmHg	113mmHg	121mmHg	116mmHg
2	131mmHg	127mmHg	132mmHg	126mmHg
3	142mmHg	130mmHg	140mmHg	134mmHg
4	112mmHg	104mmHg	113mmHg	100mmHg
5	103mmHg	103mmHg	102mmHg	105mmHg
6	118mmHg	115mmHg	116mmHg	116mmHg

* three months after the procedure

There was no significant difference between the average postoperative arterial pressure (115.3mmHg [SD: 11.3mmHg]) and the preoperative arterial pressure (121.0mmHg [SD: 13.8mmHg]) in the BCT (t=0.778, *p*=0.23). Furthermore, there was no significant difference between the postoperative arterial pressure (116.2mmHg [SD: 12.7mmHg]) and the preoperative arterial pressure (120.7mmHg [SD: 13.6mmHg]) in the LSA (t=0.592, *p*=0.28).

Animal necropsy confirmed that the aortic valves remained intact, and secure fixation and sealing of the stent grafts was achieved in all pigs (Fig 5C). Histological analysis revealed no injury at the inner walls of the target vessels (Figs 5D and 5E).

## Discussion

Stent graft treatment of aortic arch diseases is emerging as an alternative to conventional surgical repair with circulatory arrest. Inoue *et al* were the first to introduce the endovascular technique for the repair of the aortic arch.^4^ To date, reports on the use of integrated branched stent graft systems for total endovascular repair of the aortic arch have been very limited.^4–11^ Zimpfer *et al* reported an in vitro model for the endovascular exclusion of the aortic arch in pigs.^12^ In our study, we developed an improved double-branched stent graft based on Inoue’s stent graft and confirmed its feasibility for total endovascular reconstruction of the aortic arch in pigs.

### Advantages of the improved double-branched stent graft

Almost all aortic arch dissection and aneurysms involving supra-arch branch vessels can be treated with this method. Furthermore, our stent graft was introduced into the aortic arch through the infrarenal aorta, which is expected to generate less injury than a stent graft introduced through the carotid artery.^7^ According to the preoperative arteriography, the mean diameter of the common carotid arteries in these six pigs was 3.41mm (SD: 0.23mm). It is impossible to introduce an 18Fr graft system or a graft system with two 16Fr modular branched stents to the common carotid artery. In our opinion, even in human beings, the common carotid artery should not be the preferred approach for introducing any aorta stent graft.

Unlike fenestrated stent grafts^13–15^ and modular branched stent grafts,^9,10^ the integrated stent graft we designed was implanted as a whole entity, with the branches already attached to its main body, and the deployment and release of the integrated stent graft were accomplished in one step. This integrated configuration can minimise the risk of the disintegration.^16^ The branched section of the stent graft may increase the stability by creating a sufficient landing zone. The branched section and main body generated a stereo configuration, which could have secured the fixation and prevented the late migration of the stent graft.

### Feasibility of stent graft deployment

The pig has two branch arteries from the aortic arch, the BCT and the LSA (Fig 3). The BCT involves both common carotid arteries and the RSA. It is therefore necessary to cannulate through the LSA and RSA to introduce traction wires. The sharp angulation of porcine subclavian arteries (Fig 4B, white arrow) made it difficult for us to insert the cannula. We overcame this problem by stretching both ends of the guidewire in the catheter to a certain tension, which helped the 5Fr catheters get through the subclavian arteries easily.

Another challenge was the entanglement of the wires since this would lead to failure of deployment. To address this issue, we used a triple lumen catheter to ensure the wires were parallel. It must be noted that the traction wires were not passed through the triple lumen catheter. Instead, two guidewires were passed through this catheter and these were soon replaced by two 5Fr catheters, through which the traction wires were passed subsequently (Fig 3). This technique can be expanded to endovascular repair of the aortic arch with a triple-branched stent graft. A quadruple lumen catheter should be applied for triple-branched stent graft deployment instead of a triple lumen catheter.

### Complications

Inoue *et al* reported embolic cerebrovascular accident as the major complication in their initial experience of branched stent grafts.^4,5^ In our study, no embolic cerebrovascular events were observed. Our double-branched stent graft implantation therefore appears safe in terms of the risk of an embolic cerebrovascular accident in healthy animal models.

It has been proposed that the manipulation of pulling stiff branches into torturous vessels is a highly traumatic procedure. We adopted several strategies to minimise injury. First, the protective caps around the branches prevented the inner surface of the target vessels from being scraped. Second, when manipulating the traction wires, the catheters outside provided protection to the inner surface of the vessel. Third, the inner sheath provided a smooth surface for the folded stent graft, which allowed it to be delivered to the aortic arch without injury. Our postoperative histology examination revealed no intimal injury at the target vessels, indicating that introduction of the branches of the stent graft into the target vessels leads to minimal vascular injury.

During the release of the graft, a parachute-like structure formed, preventing the ejection of blood from the ventricle to the distal aorta. This disappears only when the graft is fully released. However, owing to ventricular contraction, blood flow pushes the partially released graft and causes distortion or shift of the graft. To overcome this, we adopted several measures: first, the blood pressure was decreased briefly by 20–30% and then recovered after the complete release of the graft; second, the time required for the release of the graft was minimised to avoid the push of blood flow on the graft; and third, the graft was set about 1cm ahead of the predicted position before delivery to allow for a 1cm error of shift of the graft during the release of the graft.

Another issue concerns blood supply to the brain after stent graft insertion. To evaluate the blood supply to the brain and upper extremities, the pre and postoperative blood pressure was measured in the BCT and LSA. No significant difference was found between pre and postoperative arterial pressure in either of the supra-arch branch arteries, suggesting no serious ischaemia of the brain or upper extremities (Table 2).

### Limitations

Several limitations of the present study should be pointed out. We used healthy pigs, whose aortas were free of atherosclerosis or significant enlargement. Furthermore, the vessel anatomy of pigs and humans is partially different. This model can hardly simulate a diseased human aorta. In addition, we did not examine the blood parameters and some subtle biological effects of the procedure may have been overlooked. These limitations will be addressed in our future investigations.

## Conclusions

We have developed an integrated double-branched stent graft system for the aortic arch and its supra-arch branch arteries. The application of this graft system to total endovascular repair of the aortic arch is feasible and reliable. We are considering future studies that are based on proper animal models of aortic disease. In our preliminary studies, the model of descending aorta dissection has been achieved successfully in animals.^17^ In principle, the application of this technique can be expanded to endovascular repair of the total aortic arch, leading to a wide range of therapeutic indications.^4,5,18^ The long-term feasibility, durability and safety of the improved integrated double-branched stent graft remain to be addressed in future studies.

## Funding

This work was supported by a grant from the National High Technology Research and Development Programme of China (grant number 2006AA02Z4E2) and The Youth Innovation Fund of Fujian Province (grant number 2011J05088).
